# Different Coactivator Recruitment to Human PPARα/δ/γ Ligand-Binding Domains by Eight PPAR Agonists to Treat Nonalcoholic Fatty Liver Disease

**DOI:** 10.3390/biomedicines12030624

**Published:** 2024-03-11

**Authors:** Shotaro Kamata, Akihiro Honda, Nonoka Kashiwagi, Ayumi Shimamura, Sayaka Yashiro, Yuna Komori, Aoi Hosoda, Noriyuki Akahoshi, Isao Ishii

**Affiliations:** Department of Health Chemistry, Showa Pharmaceutical University, Machida 194-8543, Tokyo, Japan

**Keywords:** coactivator, dual/pan agonist, gene expression, nonalcoholic fatty liver disease, nuclear receptor, peroxisome proliferator-activated receptor, PPARγ coactivator-1α, PPAR response element, time-resolved fluorescence resonance energy transfer, transcription factor

## Abstract

Three peroxisome proliferator-activated receptor subtypes, PPARα, PPAR(ß/)δ, and PPARγ, exert ligand-dependent transcriptional control in concert with retinoid X receptors (RXRs) on various gene sets harboring PPAR response elements (PPREs) in their promoter regions. Ligand-bound PPAR/RXR complexes do not directly regulate transcription; instead, they recruit multiprotein coactivator complexes to specific genomic regulatory loci to cooperatively activate gene transcription. Several coactivators are expressed in a single cell; however, a ligand-bound PPAR can be associated with only one coactivator through a consensus LXXLL motif. Therefore, altered gene transcription induced by PPAR subtypes/agonists may be attributed to the recruitment of various coactivator species. Using a time-resolved fluorescence resonance energy transfer assay, we analyzed the recruitment of four coactivator peptides (PGC1α, CBP, SRC1, and TRAP220) to human PPARα/δ/γ-ligand-binding domains (LBDs) using eight PPAR dual/pan agonists (bezafibrate, fenofibric acid, pemafibrate, pioglitazone, elafibranor, lanifibranor, saroglitazar, and seladelpar) that are/were anticipated to treat nonalcoholic fatty liver disease. These agonists all recruited four coactivators to PPARα/γ-LBD with varying potencies and efficacy. Only five agonists (bezafibrate, pemafibrate, elafibranor, lanifibranor, and seladelpar) recruited all four coactivators to PPARδ-LBD, and their concentration-dependent responses differed from those of PPARα/γ-LBD. These results indicate that altered gene expression through consensus PPREs by different PPAR subtypes/agonists may be caused, in part, by different coactivators, which may be responsible for the unique pharmacological properties of these PPAR agonists.

## 1. Introduction

The number of patients with nonalcoholic fatty liver disease (NAFLD)/nonalcoholic steatohepatitis (NASH) has increased to one-third of the global population [1]. Some patients further develop cirrhosis or hepatocellular carcinoma (HCC), and the treatment of NAFLD (through bariatric surgery) could reduce the incidence of HCC [2]; however, no effective drugs are currently available [3]. The intimate link between NAFLD and metabolic disorders has resulted in the renaming of NAFLD as “metabolic-dysfunction-associated fatty liver disease (MAFLD) [4]”, and drugs targeting various facets of NAFLD-associated metabolic dysfunctions (e.g., enhanced fatty acid synthesis, lipotoxicity, inflammation, and fibrosis) have been developed; however, most have been withdrawn because of their serious side effects or lack of therapeutic effects [3,5]. Of these, peroxisome proliferator-activated receptor (PPAR) agonists remain the most promising. PPARs belong to the 1C class of the nuclear receptor (NR) superfamily and are ligand-activated transcription factors (TFs) that regulate the expression of multiple gene sets involved in metabolism [6,7]. Three cognate subtypes, namely, PPARα (NR1C1), PPARδ (also known as PPARß; NR1C2), and PPARγ (NR1C3), have been identified in mammals [8]. PPARα regulates lipid and glucose metabolism through the direct transcriptional control of genes involved in peroxisomal/mitochondrial oxidation, fatty acid uptake, and triglyceride catabolism [9]. PPARδ controls energy metabolism, cell survival/differentiation, and inflammation [10], whereas PPARγ is a master regulator of adipogenesis and a potent modulator of systemic lipid metabolism and insulin sensitivity [11]. Although these PPARs share anti-inflammatory activities, they are distinguished by their varying effects on lipid/glucose metabolism [12]. Based on these data, several clinical trials using PPAR dual/pan agonists against NAFLD are currently ongoing [7].

PPARs are located in the nucleus, heterodimerized with retinoid X receptors (RXRs), and bound to the cis-acting regulatory region (i.e., PPAR response element: PPRE) upstream of the target genes. Unliganded PPAR/RXR is bound to corepressor complexes, including nuclear receptor corepressor (NCoR), and silencing mediator of retinoic acid and thyroid hormone receptor (SMRT), which recruit histone deacetylases (HDACs) to suppress target gene transcription [13]. The activation of PPAR by ligands induces a conformational change; it releases the corepressor complexes and, instead, recruits coactivator complexes to the promoter region (containing several PPRE types [14]) of target genes to initiate transcription [13]. Thus far, hundreds of coactivators, corepressors, and coregulators have been identified for a total of 48 NRs in humans [15]. Some coactivators, including PPARγ coactivator-1α/ß (PGC1α/ß), cyclic AMP responsive element binding protein [CREB]-binding protein (CBP), steroid receptor coactivator family 1/2/3 (SRC1/2/3), and thyroid hormone receptor-associated protein 220 (TRAP220), are recruited through PPAR activation [13,16]. Each coactivator appears to be required for the regulation of a subset of the genes that are direct targets of a single TF. Different gene targets of a specific TF may require different sets of coactivators for regulation by the TF in a particular cell type [15].

In this study, we examined the preferential recruitment of four representative coactivator peptides (PGC1α, CBP, SRC1, and TRAP220) to human PPARα/δ/γ-ligand-binding domains (LBDs) by eight PPAR dual/pan agonists (bezafibrate, fenofibric acid, pemafibrate, pioglitazone, elafibranor, lanifibranor, saroglitazar, and seladelpar), all of which are expected to have activity in NAFLD [7]. We used a time-resolved fluorescence resonance energy transfer (TR-FRET) assay to detect the direct physical interactions between PPARα/δ/γ-LBDs and the coactivators in a cell-free system to evaluate the ligand activities [17,18,19,20]. Our results indicate that different PPAR agonists recruit the four coactivator peptides to each of PPARα/δ/γ-LBDs at altered potencies and efficacies to exert their pharmacological properties through varying patterns of gene expression.

## 2. Materials and Methods

### 2.1. Recombinant PPARα/δ/γ-LBD Expression and Purification

Human PPARα-LBD [amino acids (AAs) 200–468], PPARδ-LBD (AAs 170–441), and PPARγ-LBD (AAs 203–477 in isoform 1) were expressed as amino-terminal His-tagged proteins using a pET28a vector (Merck KgaA [Novagen], Darmstadt, Germany) in Rosetta (DE3) pLysS competent cells (Novagen). They were purified using three-step chromatography using a cobalt-based immobilized metal affinity column [TALON Metal Affinity Resin; Takara Bio, Shiga, Japan], HiTrap Q anion-exchange column [GE Healthcare, Chicago, IL, USA], and HiLoad 16/600 Superdex 75 pg gel-filtration column [GE Healthcare], as previously detailed [17,21]. After the affinity column, the His-tag was cleaved with thrombin protease (Nacalai Tesque, Kyoto, Japan).

### 2.2. Coactivator Recruitment Assay

The activation status of each PPARα/δ/γ subtype was determined using a TR-FRET assay, which was used to detect the physical interactions between His-tagged hPPARα/δ/γ-LBD proteins and four biotin-labeled coactivator peptides that had α-helical Leu-X-X-Leu-Leu (LXXLL, X: any amino acid) motifs {PGC1α [biotin-EAEEPSLLKKLLLAPANTQ (AA 137–155)], CBP [biotin-SGNLVPDAASKHKQLSELLRGGSGS (AA 56–80)], SRC1 [biotin-CPSSHSSLTERHKILHRLLQEGSPS (AA 676–700], and TRAP220 [biotin-PVSSMAGNTKNHPMLMNLLKDNPAQ (AA 631–655)]}, all of which were synthesized with GenScript (Chiyoda, Tokyo, Japan) using a LANCE Ultra TR-FRET assay (PerkinElmer, Shelton, CT, USA) [17]. A 9.5 µL aliquot of PPARα/δ/γ-LBDs [400 nM in Buffer A: 20 mM HEPES-NaOH, pH7.4, 150 mM NaCl, 1 mM EDTA, 1 mM dithiothreitol, 0.005% Tween 20, 0.1% fatty-acid-free bovine serum albumin [(BSA)], 0.5 µL of a 100× ligand solution (in DMSO), and 5 µL of biotin-coactivator peptide (1 µM in Buffer A) were mixed in a single well of a 384-well low-volume, white, round-bottom, nonbinding-surface polystyrene microplate (No. 4513, Corning, Charlotte, NC, USA). Next, 5 µL of 8 nM Eu-W1024-labeled anti-6×His antibody/80 nM ULight-Streptavidin (PerkinElmer) was added to each well, and the microplate was incubated for 2 h in the dark at room temperature. FRET signals were measured with one excitation (340/12) and two emission (615/12 and 665/12) filters using a Varioskan Flash spectral scanning multimode reader (Thermo Fisher Scientific, Waltham, MA, USA). The parameters for the measurements at 615 nm (due to Eu-W1024) and 665 nm (due to ULight-FRET) were an integration time of 200 s and a delay time of 100 µs. The 665/615 ratio was calculated and normalized to the negative control reaction using 1% DMSO. Nonlinear fitting and calculation of EC_50_ were performed using the GraphPad Prism 5 software. The coactivator recruitment is expressed as percentages of the maximal responses induced by specific PPARα/δ/γ full agonists: GW7647 (1 µM) for PPARα, GW501516 (0.1 µM) for PPARδ, and GW1929 (1 µM) for PPARγ. GW7647, GW501516, and pioglitazone were purchased from Cayman Chemical (Ann Arbor, MI, USA). Elafibranor, lanifibranor, saroglitazar, and seladelpar were purchased from ChemScene (Monmouth Junction, NJ, USA). GW1929 was purchased from Sigma-Aldrich. Bezafibrate and fenofibric acid were purchased from Fujifilm-Wako (Osaka, Japan). Pemafibrate was kindly provided by Kowa Company, Ltd. (Tokyo, Japan).

## 3. Results

### 3.1. Recruitment of the Four Coactivator Species to Each of the PPARα/δ/γ-LBDs by Selective Full Agonists (as Control Experiments)

**Figure 1:**
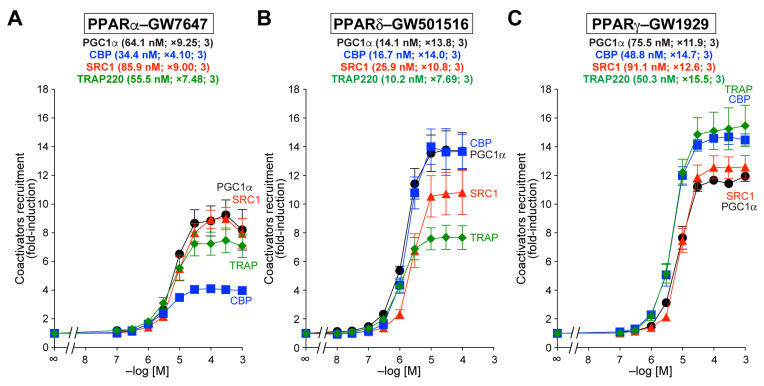
TR-FRET-based PPARα/δ/γ-LBD coactivator recruitment assay. Human PPARα/δ/γ-LBD-mediated recruitment of coactivator peptides—PGC1α (black circles), CBP (blue squares), SRC1 (red triangles), and TRAP220 (green diamonds)—was induced with selective PPAR agonists—GW7647 for PPARα (**A**), GW501516 for PPARδ (**B**), and GW1929 for PPARγ (**C**)—in a concentration-dependent manner. The data (fold-induction of basal levels) are the means ± SE of three independent experiments with duplicate samples. The averages of the calculated EC_50_ values and fold-induction and the numbers of samples are serially shown in parentheses. The maximal responses at 1 µM (**A**,**C**) or 0.1 µM (**B**) were used as 100% responses in Figure 2, Figure 3 and Figure 4.

A previous RNA sequencing study on 27 major human organs revealed distinct but generally overlapping patterns for the expression of PPARα/δ/γ and the four coactivators (Table 1) [22], suggesting that some PPAR subtypes and coactivators are expressed in a single parenchymal cell. However, few studies have been performed to comparatively analyze the physical interaction between each PPARα/δ/γ and different coactivator species in cells or cell-free systems. We recently reported the recruitment of both PGC1α and SRC1 peptides to PPARα/δ/γ-LBDs by fibrates (bezafibrate, fenofibric acid, and pemafibrate) and candidate anti-NAFLD PPAR agonists (pioglitazone, elafibranor, lanifibranor, saroglitazar, and seladelpar) using a TR-FRET assay [19,20]. We used Buffer B (10 mM HEPES-NaOH, pH 7.4, 150 mM NaCl, 0.005% Tween 20, and 0.1% fatty acid-free BSA) based on a PerkinElmer protocol for PPARα/γ-LBDs and Buffer C (50 mM HEPES-NaOH, pH 7.4, 50 mM KCl, 1 mM EDTA, 0.5 mM dithiothreitol, and 0.1% fatty acid-free BSA according to Drake et al. [23]) for PPARδ-LBD because we failed to detect the recruitment of PGC1α to PPARδ-LBD with the PPARδ full agonist GW501516 in Buffer B. In the present study, we optimized the assay buffer (Buffer A) so that equivalent levels of activation [maximal fold-induction from basal (no ligand) levels and EC_50_ values] were observed on the four coactivators with each full agonist: GW7647 (×4.10–9.25 and 34.4–85.9 nM) for PPARα, GW501516 (×7.69–14.0 and 10.2–25.9 nM) for PPARδ, and GW1929 (×11.9–15.5 and 48.8–91.1 nM) for PPARγ (Figure 1A–C, respectively). The maximal response (fold-induction) for each GW compound was expressed as 100% in the following experiments.

### 3.2. Recruitment of the Four Coactivators to PPARα-LBD by the Eight Agonists

**Figure 2:**
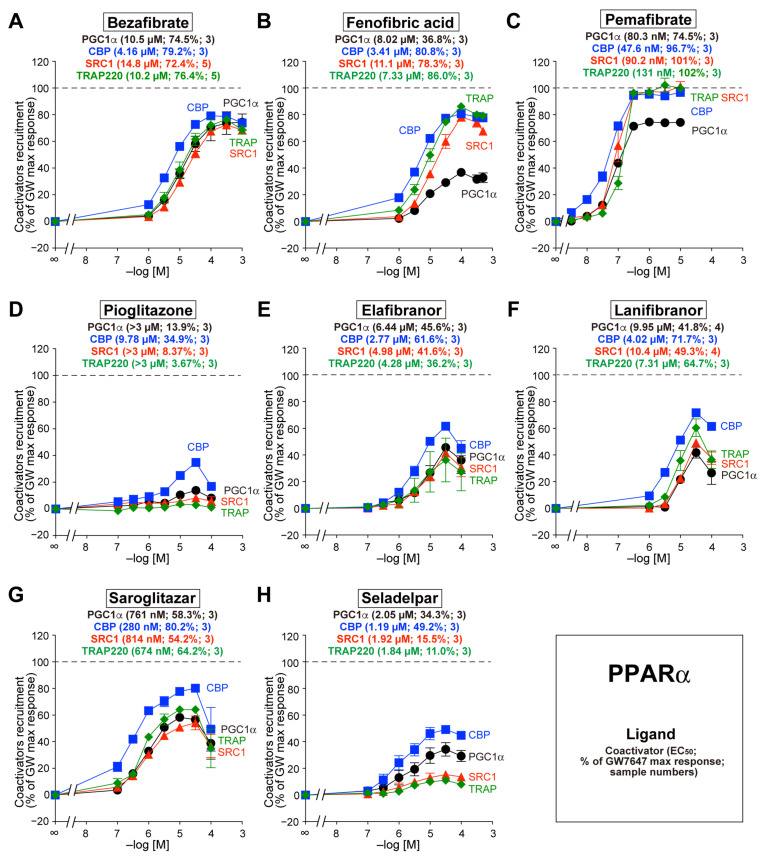
TR-FRET-based PPARα-LBD coactivator recruitment by eight PPAR dual/pan agonists. Human PPARα-LBD-mediated recruitment of coactivator peptides—PGC1α (black circles), CBP (blue squares), SRC1 (red triangles), and TRAP220 (green diamonds)—was induced by the PPAR dual/pan agonists bezafibrate (pan agonist; (**A**)), fenofibric acid (α/γ dual agonist; (**B**)), pemafibrate (pan agonist; (**C**)), pioglitazone [(α/)γ agonist; (**D**)], elafibranor (pan agonist; (**E**)), lanifibranor (pan agonist; (**F**)), saroglitazar (α/γ dual agonist; (**G**)), and seladelpar (pan agonist; (**H**)). The maximal response induced by 1 µM GW7647 (Figure 1A) was designated as the 100% response. The data are the means ± SE of 3–5 independent experiments with duplicate samples. The averages of the calculated EC_50_ values, percentage responses, and the numbers of samples are shown in parentheses.

Bezafibrate recruited all four coactivators to PPARα-LBD to a similar extent (72.4–79.2%) with a similar EC_50_ (4.16–14.8 µM) (Figure 2A). In contrast, the recruitment of PGC1α by fenofibric acid and bezafibrate was less pronounced than that of other coactivators (Figure 2B and Figure 2C, respectively). The maximal response induced by the other five agonists was generally smaller than those induced by the three vibrates, and the recruitment of CBP was the most evident (Figure 2D–H).

### 3.3. Recruitment of the Four Coactivators to PPARδ-LBD by the Eight Agonists

**Figure 3:**
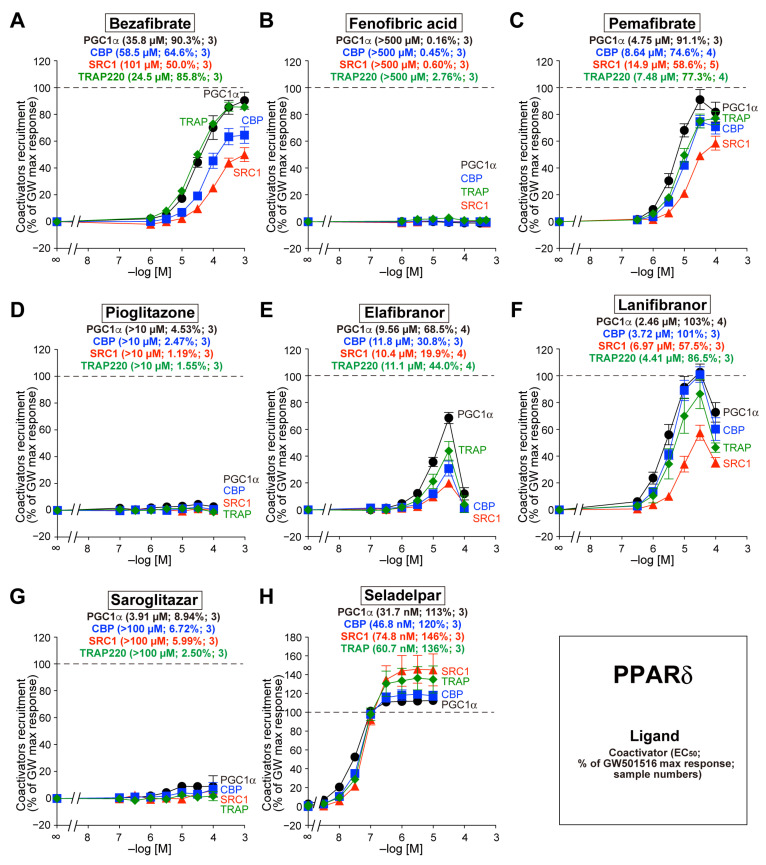
TR-FRET-based PPARδ-LBD coactivator recruitment by eight PPAR dual/pan agonists. Human PPARδ-LBD-mediated recruitment of coactivator peptides—PGC1α (black circles), CBP (blue squares), SRC1 (red triangles), and TRAP220 (green diamonds)—was induced by the PPAR dual/pan agonists bezafibrate (pan agonist; (**A**)), fenofibric acid (α/γ dual agonist; (**B**)), pemafibrate (pan agonist; (**C**)), pioglitazone [(α/)γ agonist; (**D**)], elafibranor (pan agonist; (**E**)), lanifibranor (pan agonist; (**F**)), saroglitazar (α/γ dual agonist; (**G**)), and seladelpar (pan agonist; (**H**)). The maximal responses induced by 0.1 µM GW501516 (Figure 1B) were used as the 100% responses. The data are the means ± SE of 3–5 independent experiments with duplicate samples. The averages of the calculated EC_50_ values, percentage responses, and the numbers of samples are shown in parentheses.

Bezafibrate also recruited all four coactivators to PPARδ-LBD, but the potencies and efficacy differed from those of PPARα-LBD. Bezafibrate recruited PGC1α and TRAP220 more effectively than CBP or SRC1 (Figure 3A). Fenofibric acid, pioglitazone, and saroglitazar did not recruit any coactivators to PPARδ-LBD, whereas pemafibrate, elafibranor, lanifibranor, and seladelpar were effective (Figure 3B–H). Of note, seladelpar acted as a full agonist of PPARδ (113–146%) with EC_50_ values as low as 31.7–74.8 nM (Figure 3H). 

### 3.4. Recruitment of the Four Coactivators to PPARγ-LBD by the Eight Agonists

**Figure 4:**
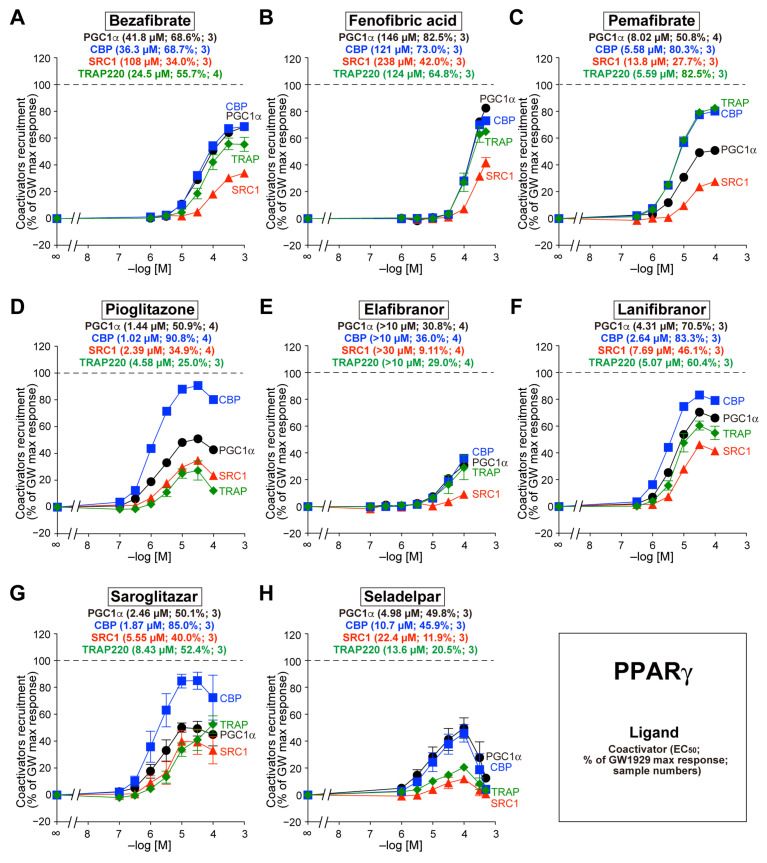
TR-FRET-based PPARγ-LBD coactivator recruitment by eight PPAR dual/pan agonists. Human PPARγ-LBD-mediated recruitment of coactivator peptides—PGC1α (black circles), CBP (blue squares), SRC1 (red triangles), and TRAP220 (green diamonds)—was induced by the PPAR dual/pan agonists bezafibrate (pan agonist; (**A**)), fenofibric acid (α/γ dual agonist; (**B**)), pemafibrate (pan agonist; (**C**)), pioglitazone [(α/)γ agonist; (**D**)], elafibranor (pan agonis; (**E**)), lanifibranor (pan agonist; (**F**)), saroglitazar (α/γ dual agonist; (**G**)), and seladelpar (pan agonist; (**H**)). The maximal responses induced by 1 µM GW1929 (Figure 1C) were used as the 100% responses. The data are the means ± SE of 3–4 independent experiments with duplicate samples. The averages of the calculated EC_50_ values, percentage responses, and the numbers of samples are shown in parentheses.

Bezafibrate also recruited all four coactivators to PPARγ-LBD, but its potencies and efficacies differed from those of PPARα/δ-LBD. For example, bezafibrate recruited PGC1α/CBP/TRAP220 more effectively than SRC1 (Figure 4A). The other seven agonists also recruited all coactivators to PPARγ-LBD but with varying potencies and efficacy (Figure 4B–H).

## 4. Discussion

Previous transcriptome (microarray) analyses demonstrated altered patterns of gene expression in cells or animals administered different PPAR agonists: bezafibrate, fenofibrate, and WY-14643 [24], gemfibrozil and clofibrate [25], fenofibrate and pemafibrate [26,27], and even endogenous long-chain fatty acids (e.g., linoleic/linolenic acids vs. palmitic/oleic acids) [28]. This may be attributed, in part, to the PPAR subtypes on which they act; however, ligand binding to PPARs is not sufficient for transcriptional regulation, and the subsequent recruitment of multiprotein coactivator complexes is indispensable. Ligand binding to the ligand-binding pocket of PPAR induces a conformational change and releases corepressor complexes such as NCoR and SMRT, and a helix 12 (H12) is stabilized by the activation function 2 (AF-2) surface of PPAR [17,29,30]. The stabilized H12, H3, and H4 consist of a hydrophobic core that accepts an LXXLL motif, which is conserved in coactivators [31]. This pocket only accepts a single LXXLL motif [29], as determined by the analyses of cocrystals using X-ray crystallography [17], even though several coactivators are expressed within a cell (Table 1), and some coactivators contain multiple LXXLL motifs. The recruited coactivator complex initiates the transcription of its target genes through the acetylation or methylation of histones, chromatin remodeling (due to helicase activity), and the recruitment of other coregulators [16]. Therefore, the differential recruitment patterns of coactivator complexes for different PPAR subtypes/agonists may affect the gene expression profile. We determined how each PPAR agonist recruited different coactivators to PPARα/δ/γ-LBDs using a highly sensitive cell-free TR-FRET system. Although numerous coactivators were recruited upon PPAR activation under physiological conditions [13], four representative coactivators (PGC1α, CBP, SRC1, and TRAP220) containing LXXLL motifs were selected for the comparative analyses. 

Some coactivators exhibit histone acetyltransferase (HAT) activity, which acetylates Lys residues on the amino-terminal tails of histones to disrupt nucleosomes and initiate transcription. Other types recruit multiprotein complexes with HAT activity [13,16]. Transcriptional coactivator complexes, which are composed of homologous CBP and p300 (adenovirus E1A-associated 300-kDa protein), are key regulators of RNA-polymerase II-mediated transcription and exhibit HAT activity [32]. The human CBP gene encodes a 265-kDa protein consisting of 2442 AAs, and the human p300 gene encodes a 265-kDa protein consisting of 2414 AAs; these contain two and three LXXLL motifs, respectively. The high-molecular-weight CBP and p300 proteins are common coactivators for multiple transcriptional factors, and both are essential for mouse embryonic development [33,34]. CBP (and PGC1α, SRC1, etc.) was identified as part of a transcriptionally active PPARα-interacting cofactor (PRIC) complex [35], and p300 was shown to interact with mouse PPARα (but not retinoic acid receptor γ [RARγ] or RXRα) upon ligand binding and to enhance its transcriptional activity [36]. Overexpression of CBP and p300 is indispensable for the adipogenic differentiation of 3T3-L1 cells through PPARγ regulation [37]. CBP-heterozygous (CBP^+/−^) mice exhibited markedly reduced weight of white adipose tissues but not of other tissues, whereas their insulin sensitivity and glucose tolerance were increased. The expression of PPARα and its target genes that control lipid metabolism was induced in the skeletal muscle, liver, and brown adipose tissue (BAT) of CBP^+/−^ mice [38], although the involvement of PPARs in their lipodystrophic phenotypes remains to be clarified. In humans, the upregulation of p300 mRNA and protein was found in the majority of HCC tissues [39], and CBP/p300-mediated acetylation of H3K18 and H3K27 was increased in HCC tissues compared with that in surrounding non-cancer tissues [40]. Moreover, a p300 inhibitor (B029-2) suppressed the proliferation of Huh7 and Hep3B cells by reducing the acetylated H3K18/H3K27 levels and inhibiting mRNA expression of phosphoserine phosphatase (PSPH) and deoxythymidylate kinase (DTYMK) [40]. Therefore, the CBP/p300 recruitment by PPAR subtypes/agonists could be undesirably implicated in HCC pathogenesis/progression. To our knowledge, our results are the first demonstration of ligand-dependent CBP recruitment via PPARδ, which was found to be activated with GW501516, bezafibrate, elafibranor, lanifibranor, and seladelpar (Figure 1B, Figure 3A, Figure 3C, Figure 3E, Figure 3F, and Figure 3H, respectively). 

The p160/SRC family of coactivators—SRC1 (NCOA1), SRC2 (NCOA2/TIF2/GRIP1), and SRC3 (NCOA3/pCIP/RAC3/ACTR/AIB1/TRAM-1)—are among the first cloned coactivators based on their ligand-dependent human progesterone receptor (NR) recruitment activity [41]. These coactivators are involved in various aspects of gene expression regulation, including transcriptional initiation, coregulator recruitment, RNA splicing, posttranslational modifications of NRs/coregulators, and translation [16,42,43]. The human SRC1 gene encodes a 157-kDa protein consisting of 1441 amino acids, including 7 LXXLL motifs. Its amino-terminal basic helix–loop–helix–Per/ARNT/Sim (bHLH-PAS) domain facilitates protein–protein interactions with other coregulator complexes and TFs and contains a canonical nuclear localization signal [42,43]. Mice lacking SRC1, SRC2, or SRC3 (SRC1^−/−^, SRC2^−/−^, or SRC3^−/−^) are viable, fertile, and exhibit PPARα-mediated gene expression and different physiological responses when challenged with PPARα agonists. In contrast, SRC1^−/−^/SRC2^−/−^ and SRC1^−/−^/SRC3^−/−^ double-null mice are embryonically lethal, suggesting that SRC1/2/3 contains both redundant and distinct biological functions [13]. SRC1 and SRC3 were shown to be upregulated in 47.5% of human HCC specimens, and the downregulation of SRC1 decreased the proliferation of various human HCC cell lines and impaired xenograft tumor maintenance in nude mice [44]. Alternatively, an imbalanced expression pattern of SRC1 and SRC3 compared with that in the normal liver (decreased SRC1 and increased SRC3) might be involved in the occurrence of HCC [45]. Therefore, the altered recruitment of SRC1 by PPAR subtypes/agonists could affect hepatic carcinogenesis in either direction.

The PGC1 family of coactivators (PGC1α, PGC1ß, and PRC) is a key player in the regulation of energy metabolism [46,47]. Unlike the CBP/p300 and p160/SRC families that possess intrinsic HAT activity, the PGC1 family lacks HAT activity but shares highly conserved amino-terminal domains that recruit HAT proteins, such as CBP/p300 and SRC1 [48]. The human PGC1α gene encodes a 91-kDa protein consisting of 798 amino acids that contain a single LXXLL motif at the amino-terminus and a single RNA recognition motif at the carboxyl-terminus [49]. PGC1α is the master regulator of adaptative thermogenesis and mitochondrial biogenesis, and it is induced by a high energy demand and regulates overlapping gene expression programs [46,47]. Both PGC1α and PGC1ß are highly expressed in tissues with high energy requirements and mitochondrial content, including the skeletal muscle, liver, heart, and BAT, whereas PRC exhibits comparable expression across different tissues [47]. PGC1α is induced by different physiological (e.g., fasting, exercise, and cold exposure) and pharmacological cues [47,50] and is recruited by all PPAR subtypes [17,18,19,20,47], as observed in the present study (Figure 2, Figure 3 and Figure 4). Genetic studies revealed that the PGC1α gene rs8192678 G>A (Gly482Ser) polymorphism was associated with the severity of NAFLD features in severely obese Taiwanese patients [51] or those from the Chinese Han population [52]. The regulation of PGC1α via PPARs could be a rheostat of NAFLD progression. 

TRAP220—also known as PPAR-binding protein (PBP), vitamin D receptor-interacting protein 205 (DRIP205), and mediator 1 (MED1)—directly binds to PPARα, RARα, RXR, and other NRs. The human TRAP220 gene encodes a 168-kDa protein consisting of 1581 amino acids that does not exhibit HAT activity but contains two LXXLL motifs and serves as an anchor for the multisubunit “Mediator” complex. The deletion of the carboxyl-terminal AF-2 domain from PPARγ interferes with the interaction between TRAP220 and PPARγ, and a truncated form of TRAP220 acts as a dominant–negative repressor [16]. Mice lacking TRAP220 are embryonically lethal, and liver-specific deletion of TRAP220 in mice resulted in the near abrogation of PPARα ligand-induced responses [16]. Furthermore, glutathione S-transferase (GST) pull-down assays revealed that PPARδ interacts with TRAP220, SRC1/2/3, NCoR, and SMRT in the absence of ligands [53]. Therefore, TRAP220 may interact with all PPAR subtypes [14], which is supported by the results of the present study (Figure 1, Figure 2, Figure 3 and Figure 4). The expression of TRAP220 was increased in the livers of NASH patients and mice and was positively correlated with transforming growth factor ß (TGF-ß) signaling and profibrotic factors [54]. In addition, the expression of miR-146a, which directly targets TRAP220, was significantly decreased in the livers of high-fat-diet-fed and ob/ob mice [55]. The functional regulation of TRAP220 by PPAR subtypes/agonists may also affect the progression of NAFLD/NASH.

**Figure 5:**
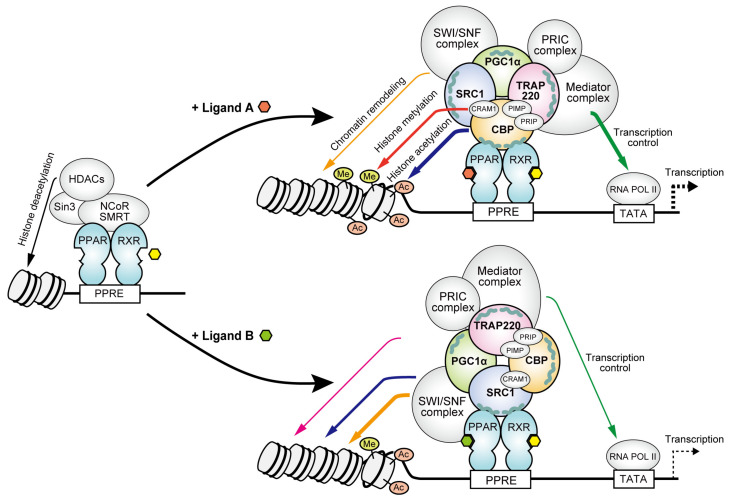
The different coactivator recruitment may alter gene expression profiles. The PPARα/δ/γ and PPAR ligand combination determined the coactivator species (e.g., PGC1α, CBP, SRC1, and TRAP220) with which they interacted via the LXXLL motif, thereby determining the orientation of the entire multiprotein coregulator complexes, including PRIC (which remodels chromatin through histone displacement and nucleosome sliding with helicase activity), SWI/SNF (which mobilizes the nucleosome with ATPase activity), and Mediator (which anchors TRAP220 and facilitates recruitment and activation of the RNA polymerase-II-associated basal transcription machinery) complexes, and resulting in the altered transcription of target genes.

Taking these results together, we observed varying concentration-dependent recruitment of four major coactivator peptides toward PPARα/δ/γ-LBDs by eight PPAR agonists. The approximate potency order is summarized in Table 2. PPARα/δ/γ favored CBP, PGC1α, and CBP/PGC1α, respectively, in general, but there were some exceptions, such as bezafibrate, which slightly favored TRAP220 over PGC1α for PPARδ. Our proposed model is illustrated in Figure 5. The PPARα/δ/γ and PPAR ligand combination (as well as the combination of RXRs and RXR ligands) determined the coactivator species with which they interacted via the LXXLL motif, thereby determining the orientation of the whole multiprotein coregulator complexes, which may have largely affected the transcription of their target genes. This may be particularly important for the clinical application of PPAR dual/pan agonists that exhibit both common and distinct pharmacological properties.

## Figures and Tables

**Table 1:** Tissue-specific expression (FPKM values) of PPAR and its coactivator genes in major human organs.

	PPAR Transcripts			Coactivator Transcripts			
	*PPARA*	*PPARD*	*PPARG*	*PGC1A*	*CBP*	*SRC1*	*TRAP220*
Gene ID ^1^	5465	5467	5468	10891	1387	8648	5469
FPKM values							
**adrenal**	3.35	4.34	0.349	0.985	4.09	8.79	4.35
**appendix**	2.20	7.55	0.797	0.181	7.15	8.66	7.69
**bone marrow**	0.492	2.04	0.0962	0.00309	11.6	6.44	5.34
**brain**	2.53	7.43	0.165	1.85	6.46	17.4	5.10
**colon**	5.88	7.77	3.61	2.11	5.63	8.12	5.62
**duodenum**	7.53	3.85	1.75	2.26	4.36	10.8	4.14
**endometrium**	2.15	6.20	0.490	0.307	10.3	10.0	7.21
**esophagus**	3.39	9.75	0.559	0.944	5.21	10.3	5.33
**fat**	3.91	5.05	18.9	0.696	6.87	8.08	5.53
**gall bladder**	2.57	6.57	1.11	0.822	6.55	9.84	5.80
**heart**	8.09	3.91	0.557	5.82	3.27	6.17	3.50
**kidney**	11.9	3.55	1.14	7.60	5.82	9.72	5.23
**liver**	6.43	1.35	1.00	5.97	3.38	4.28	2.71
**lung**	1.80	6.07	2.03	1.35	6.51	9.06	5.56
**lymph node**	1.53	5.37	0.370	0.0698	6.65	8.50	8.09
**ovary**	4.89	7.48	0.966	0.343	12.1	13.4	6.58
**pancreas**	0.893	0.845	0.0542	0.269	1.96	2.15	1.04
**placenta**	1.79	13.0	3.48	0.0421	6.31	7.72	5.84
**prostate**	2.91	6.53	0.419	0.983	6.03	8.15	4.96
**salivary gland**	1.56	2.28	0.193	3.66	2.84	3.75	1.98
**skin**	3.22	7.33	0.0981	0.173	6.47	7.69	4.80
**small intestine**	7.96	5.07	1.22	1.94	4.81	10.0	4.26
**spleen**	1.98	6.75	1.07	0.200	9.28	8.99	6.61
**stomach**	1.98	7.95	2.89	0.862	5.12	5.47	4.28
**testis**	0.989	5.43	0.801	1.68	12.7	14.6	7.22
**thyroid**	3.97	10.7	1.16	5.52	8.11	7.65	6.88
**urinary bladder**	2.76	5.30	4.24	0.279	5.75	8.39	6.14

RNA sequencing was performed on tissue samples from 95 human individuals representing 27 different tissues [22], and the values of the fragments per kilobase of exon model per million mapped reads (FPKM) were calculated [22]. The use of human tissue samples was approved by the Uppsala Ethical Review Board (Reference #2011/473) [22]. ^1^ Data are available from the Gene Database of the National Library of Medicine with those Gene IDs (https://www.ncbi.nlm.nih.gov/gene; accessed on 13 February 2024).

**Table 2:** Approximate potency order in the recruitment of four coactivators by the PPAR subtype/agonist combinations.

	PPARα	PPARδ	PPARγ
**Bezafibrate**	CBP>PGC=TRAP>SRC	TRAP>PGC>CBP>SRC	PGC=CBP=TRAP>SRC
**Fenofibric acid**	CBP>TRAP>PGC>SRC	No activation	PGC=CBP=TRAP>SRC
**Pemafibrate**	CBP>PGC=SRC>TRAP	PGC>CBP=TRAP>SRC	CBP=TRAP>PGC>SRC
**Pioglitazone**	CBP>>>PGC=SRC=TRAP	No activation	CBP>PGC>SRC>TRAP
**Elafibranor**	CBP>PGC=SRC=TRAP	PGC>TRAP>CBP>SRC	PGC=CBP=TRAP>SRC
**Lanifibranor**	CBP>TRAP>PGC=SRC	PGC>CBP>TRAP>SRC	CBP>PGC>TRAP>SRC
**Saroglitazar**	CBP>TRAP>PGC=SRC	Faint activation	CBP>PGC>TRAP=SRC
**Seladelpar**	CBP>PGC>SRC=TRAP	PGC>CBP>SRC=TRAP	PGC=CBP>TRAP>SRC

Coactivator recruitment potencies were evaluated based on EC_50_ values. PGC, PGC1α; SRC, SRC1; TRAP, TRAP220.

## Data Availability

Data are contained within the article.
